# A large scale 16S ribosomal RNA gene amplicon dataset of hand, foot and mouth patients and healthy individuals

**DOI:** 10.1038/s41597-023-01953-2

**Published:** 2023-01-21

**Authors:** Xiaoying Guo, Min Yan, Dongyi Huang, Siyi Chen, Dantao Zhang, Zhifeng Li, Xingfen Yang, Wei Wu

**Affiliations:** 1 School of Public Health, Southern Medical University, Guangzhou, China; 2 Guangdong Provincial Institute of Public Health, Guangdong Provincial Center for Disease Control and Prevention, Guangzhou, China

**Keywords:** Infectious-disease epidemiology, Risk factors

## Abstract

There is evidence linking hand, foot and mouth disease (HFMD) to gut microbiota dysbiosis, and this relationship was corroborated in a large HFMD patient population in our previous study. Here, we present a bacterial 16S rRNA gene dataset from faecal samples of 713 individuals (254 HFMD patients, 459 healthy controls) aged 2 to 7 years residing in Heyuan and Jiangmen counties, Guangdong Province, southern China. Microbiome analysis indicated a significant increase in genus *Prevotella*, *Cetobacterium*, and *Megamonas* was observed in patients with HFMD, whereas a large increase in genus *Bacteroides*, *Ruminococcus*, and *Faecalibacterium* were seen in the control group. We also share the bioinformatic analytical pipeline for this analysis, from data preprocessing to data filtering and amplicon sequence variant (ASV) table generation. We expect that the dataset will be reprocessed, evaluated and fully analysed with various analysis methods to further elucidate the role of the gut microbiota in HFMD development.

## Background

Thousands of microorganisms colonize the gastrointestinal (GI) tract and work in concert with the intestinal epithelial barrier to defend against pathogen invasion^1,2^. Microorganisms are highly dynamic and variable among individuals and throughout development due to environmental and individual factors, such as host genotype, diet and lifestyle^3–5^. With advances in high-throughput sequencing technologies and related bioinformatics methods, especially 16S ribosomal RNA (rRNA) gene analysis, we have been able to obtain deeper insight into the effects of intestinal microorganisms. 16S rRNA gene analysis has become the main focus of meta-taxonomic analysis and is widely used in microbiome research^6,7^.

Hand, foot, and mouth disease (HFMD) is a self-limiting disease resulting from infection with some human enteroviruses, including enterovirus A71 (EV71), coxsackievirus A16 (CVA16) and coxsackievirus A10 (CV-A10), that is prevalent in the neonatal and paediatric populations^8^. The predominant clinical manifestations of HFMD are a transient maculopapular rash and blisters or ulcers involving the hands, soles of the feet and mouth that last approximately half a week with no observable sequelae in most cases^9^. The specific pathogenesis of HFMD is not completely understood.

Although enteroviruses are the causative agents of HFMD in children, the role of the gut microbiota, constituting the intestinal microorganisms, in enterovirus pathogenesis and disease progression needs to be considered. The gut microbiota is a key component in intestinal immune responses and repairing the injured intestinal mucosa barrier to protect against pathogen invasion and maintain homeostasis^10,11^. In addition to the beneficial effects of bacteria in the gut, the significance of the gut bacterial composition in the emergence and development of certain diseases has been emphasized in some studies. The presence of harmful intestinal bacteria may induce the development of inflammatory bowel disease^12^. The intestinal microbiota of individuals with HFMD has been demonstrated to be altered in type and relative abundance of bacteria, and these alterations correlate with the severity of HFMD^13^. Whether a similar relationship exists between gut flora dysbiosis and the occurrence of HFMD is unknown. Only limited studies with very small sample sizes have focused on characteristic changes in the gut microbiota profile of children with HFMD. To further reveal the role and underlying mechanisms of intestinal flora dysbiosis in the pathogenesis of HFMD, larger population studies are needed.

Guangdong Province, an area with a high prevalence of HFMD in the Asia-Pacific region, has several designated HFMD surveillance sites, including Heyuan City and Jiangmen City. In a previous case‒control study, we enrolled 749 participants (including 262 individuals with HFMD and 487 healthy controls) from the Heyuan and Jiangmen regions and obtained faecal samples for 16S rRNA gene sequencing, followed by bioinformatics analysis for profiling. We compared the composition and variability in gut microbe communities between the different groups, among different enterovirus types and according to supplement intake. The results showed that the distribution characteristics of the intestinal flora in individuals with HFMD differed from those in healthy subjects. Compared with those in the healthy group, the relative abundances of *Prevotella* and *Streptococcus* were increased in the case group, while the relative abundances of the beneficial bacteria *Bifidobacteria* and *Faecalibacterium* were decreased. In addition, we concluded that administered synbiotics supplements potentially provide better resistance to gut dysbiosis in patients with HFMD than probiotics or prebiotics alone. Greater gut flora disturbance was observed in patients who were enterovirus A71 (EV71)-positive than in those who were coxsackievirus A16 (CAV16)-positive^14^.

We reorganized the database in order to maximize the presentation of our database and to make the dataset of higher quality. The final submitted dataset was from 713 study participants, including 459 healthy children and 254 patients with HFMD. And an analysis result of case-control study in the ratio of 1:1 was also provided. The details pertaining to the study design, sample collection, raw sequence data, bioinformatics analysis pipeline, and results of our bioinformatic analysis are provided in the subsequent sections. We expect the dataset provided here to be fully utilized and comparatively evaluated by the scientific community to provide detailed insight into the potential relationship between the gut microbiota and HFMD; understanding this relationship may contribute to improving intervention and treatment strategies for the disease.

## Methods

### Study design

All participants were selected from a previous investigation conducted by the Guangdong Institute of Public Health in 2017 that aimed to explore the distribution characteristics of gut microbiota and its related influencing factors in children with HFMD. The previous study was approved by the Ethical Review Board of Guangdong Provincial Center for Disease Control and Prevention (NO: 2016016). The legal guardians/parents of all the children were informed of the study objectives and sampling procedure and provided written informed consent prior to completing the survey. Personal identifiable information pertaining to participants was kept private. The cohort size for the study was statistically derived. Supplementary Fig. 1 depicts the overall course of the produce of this dataset.

### Participants and sampling

Two distinct centres, including Xinhui District of Jiangmen City and Longchuan County of Heyuan City in Guangdong Province were investigation sites and two local hospitals, including a children’s hospital and a county-level hospital, were selected in each investigation site to collected specimens. The case group at each investigation site was randomly selected from the local outpatient departments of hospitals, and the control group was recruited from the same hospitals or the neighbourhood of the patients. The participants were required to complete a disease risk-factor questionnaire that collected information about demographic characteristics, hygienic habits, and dietary habits in the last month. The selection criteria used for study inclusion were as follows: a) age between 2 and 7 years old; b) residency in one county or district for at least 6 months; and c) HFMD diagnosed by medical institutions in the county or district according to the Chinese Guidelines for HFMD Diagnosis and Treatment issued by the Ministry of Health in 2010, and the results were reviewed by the laboratory of Guangdong Provincial Center for Disease Control and Prevention. The exclusion criteria were as follows: a) a history of critical illness, herpetic pharyngitis or severe organic lesions; b) a disease related to the risk factors in the study, including diarrhoea, gastroenteritis, previous diagnosis of HFMD, or herpetic angina, among others; or c) having potential risk of exposure to or taking antibiotics within a month.

Participants were required to deliver faecal samples of approximately 5–8 g within 7 days of the onset of illness. At the laboratory, samples were immediately placed into a sterile tube and labelled with the child’s name, identification code and bristol classification number. The collected samples were reserved in a 4 °C refrigerator for temporary preservation within 2 h, stored in a −18 to −20 °C freezer for storage within 12 h and transferred to a −80 °C freezer after 12 h until further processing. All collection staff wore sterilized, clean, new laboratory gloves and facemasks to avoid bacterial contamination in this study.

### DNA isolation, PCR amplification and sequencing

Genomic DNA was extracted with the MagaBio Feces Genomic DNA Purification Kit (Bioer Technology, Hangzhou, China) following the manufacturer’s instructions. Subsequently, amplicons of the V4-1 hypervariable region of the bacterial 16S rRNA gene were generated using the specific primers 515 F/R806 (GTGCCAGCMGCCGCGGTAA- GGACTACHVGGGTWTCTAAT) with 12-bp barcodes. Primers were synthesized by Invitrogen (Carlsbad, CA, USA). A PCR BioRad S1000 instrument (Bio-Rad Laboratory, CA, USA) with a PCR mixture containing 25 μl of 2x Premix Taq (Takara Biotechnology, Dalian Co. Ltd., China), and 1 μl of each primer (10 μM) and 3 μl DNA (20 ng/μl) template in a total volume of 50 µl were used for bacterial amplicon amplification. The thermocycling scheme was initialization for 5 min at 94 °C; 30 cycles of denaturation for 30 s at 94 °C, annealing for 30 s at 52 °C, and extension for 30 s at 72 °C; followed by final elongation for 10 min at 72 °C. Only samples with bright primary bands between 290 and 310 bp were used in further experiments. Sequencing libraries were constructed using the NEBNext Ultra II DNA Library Prep Kit for Illumina (New England Biolabs, MA, USA) according to the manufacturer’s recommendations. Finally, the library was sequenced on the Illumina Nova6000 platform, and 250 bp paired-end reads were produced at Guangdong MagiGene Biotechnology Company (Guangdong MagiGene Biotechnology Co., Ltd., Guangzhou, China).

### Sample selection and bioinformatics analysis pipeline

**Fig. 1:**
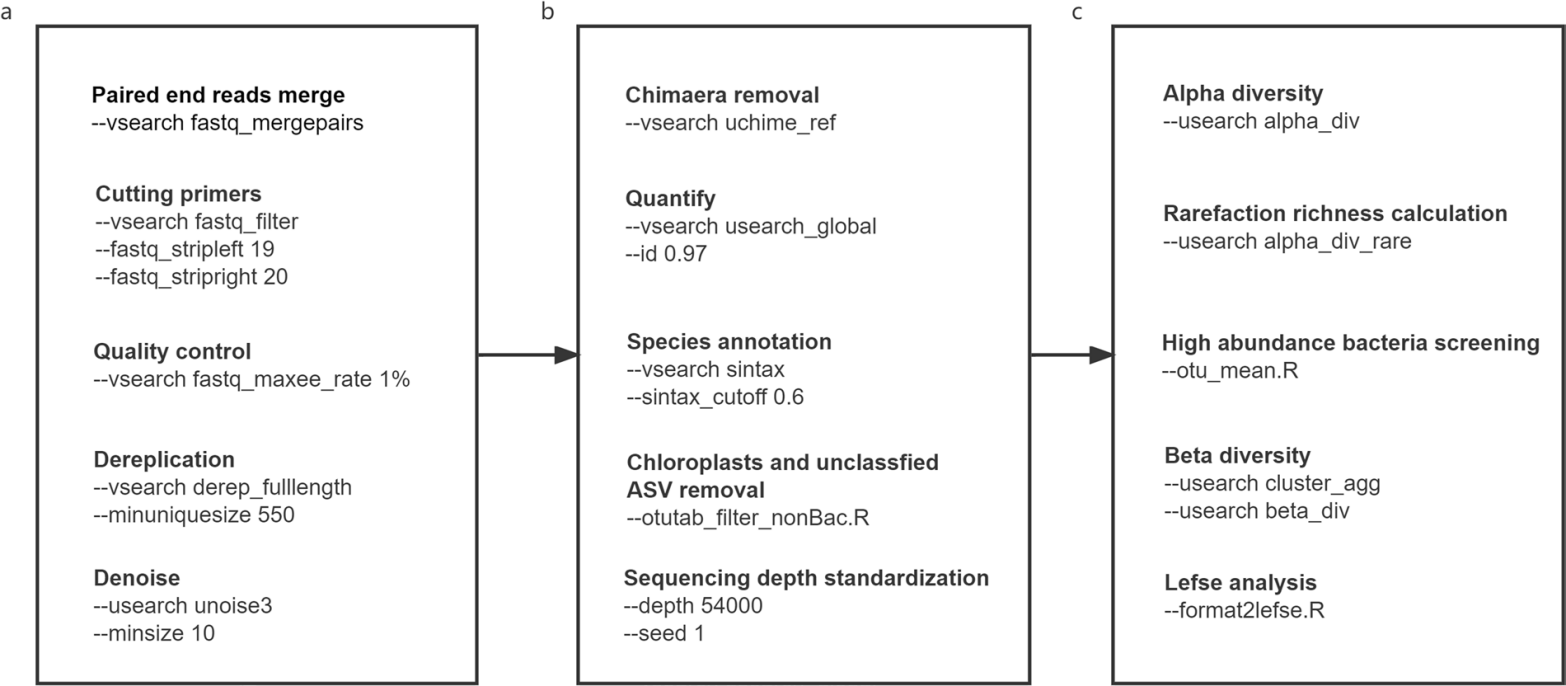
The Details of the workflow and tools used for data processing and analysis in the EasyAmplicon pipeline. (**a**) Sequences preprocessing. (**b**) Feature table construction and processing. (**c**) Species diversity and differential analysis.

A 1:1 matched case-control study among 713 participants was implemented by using SPSS 23.0 (IBM SPSS Statistics for Windows, Version 23.0, IBM Corp, Armonk, NY, USA) to minimize the effect of potentially confounding factors age and sex. 229 patients with HFMD and 229 healthy control subjects reserved after case control matching. Figure 1 depicts the overall workflow and tools used for data processing and analysis in the present study. The analysis of all samples was performed by running EasyAmplicon pipeline constructed by EHBIO Gene Technology Company (Beijing, China) and the pipeline can freely available at https://github.com/YongxinLiu/EasyAmplicon^15^. The pipeline allows analysis and visualization of microbiome data, especially 16S rDNA amplicon sequencing. Three main analysis steps of the pipeline were used in this analysis, including sequence data preprocessing, feature table construction and processing, and species diversity and differential analyses. All bioinformatic and statistical analyses were conducted in R software, version 4.1.3 (R Foundation for Statistical Computing, Vienna, Austria) unless otherwise stated. Before raw data processing, the statistics for each sample sequence were computed with the seqkit program^16^ (v0.15.0), and the results are available in Figshare File 1^17^.

Sequence merging, primer removal, quality filtering, and dereplication and denoising of amplicon sequence variants (ASVs) were performed in the preprocessing step. According to the principle of overlap between paired-end reads, the software VSEARCH version 2.15.2^18^ (fastq_mergepairs) was used to combine the forward and reverse reads. We identified and removed primer sequences at the beginning and end of the sequence using VSEARCH (fastx_filter command: fastq_stripleft 19, fastq_stripright 20) to control the maximum error rate within 1% (fastq_maxee_rate 1%). Then, the filtered reads were dereplicated by omitting population-level singletons to identify the set of unique sequences (VSEARCH derep_fulllength: minuniquesize 500). Unique sequences were denoised with the UNOISE algorithm, which controlled the ASV number to reduce the size of the dataset and achieve single-base ASV accuracy (USEARCH v10.0.240 unoise3: minsize 10)^19^. Finally, the program VSEARCH with reference mode (uchime_ref) mapping against the Ribosomal Database Project database (RDP_16 S_V18)^20^ was used for chimaera detection and removal.

In the second step of the pipeline, we constructed a feature table of ASVs abundance by mapping all the filtered reads to the list of chimaera-free ASVs, with a similarity threshold of 0.97, using VSEARCH (usearch_global). Additionally, taxonomy was annotated using the ‘VSEARCH–sintax’ command (sintax_cutoff 0.6) in combination with the RDP database (Figshare File 2). ASV_247 were removed from the dataset using ‘otutab_filter_nonBac.Rscripts’, as those were annotated as unclassified ASVs. The filtered ASVs table are displayed in Figshare File 3^17^. Eventually, to standardize the sequencing depth among the samples, the analysis with the lowest number of sequences by random resampling (depth 52000, seed 1) was performed using ‘otutab_rare.Rscripts’ with the ‘vegan’ package (v2.5–7).

Analyses involving the calculation of alpha diversity indices utilized the Shannon, Chao1, ACE, Richness, Simpson and InvSimpson indices, which are sensitive for high-abundant bacteria and can be computed by using R software with the ‘USEARCH -alpha_div’ command. To capture the contribution of low-abundant bacteria, the Tail statistic, a rank-based diversity measure was calculated as described by Li *et al*.^21^ by using custom code (Supplementary File 1). Species richness for plotting rarefaction curve was computed using USEARCH (alpha_div_rare). To analyse discrepancies between groups, dissimilarity matrices were calculated (Bray_Curtis, Euclidean, Jaccard, Manhattan, UniFrac) on the basis of the ASV tree using USEARCH (cluster_agg, beta_div), and principal coordinate analysis (PCoA) was performed. Additionally, permutational analysis of variance (PERMANOVA)^22^ was performed with the function Adonis of the ‘vegan’ package to evaluate significant differences. The identification of high-abundance (average abundance over 0.1%) bacteria in each group was conducted with ‘otu_mean. Rscript’. Linear discriminant analysis effect size (LEfSe) was performed with a Linear Discriminate Analysis (LDA) using ‘format2lefse.Rscript’ to determine the potential taxa that most likely explain the differences between the case and control groups. Finally, we combined the feature tables with annotated species information into tables at the phylum (Figshare File 4), order (Figshare File 5), class (Figshare File 6), family (Figshare File 7) and genus levels (Figshare File 8); such tables allowed further analysis of different levels of gut flora^17^. In total, raw RDP taxonomic profiles generated a total of 551 ASVs, which were annotated to 8 bacterial phyla, 15 classes, 21 orders, 38 families, and 103 genera.

## Data Records

A 16S rRNA gene raw sequencing dataset entitled 16S rRNA gene sequence data from children with hand, foot and mouth disease and healthy individuals is available for download on the Genome Sequence Archive (GSA) platform, under GSA ID CRA009110 (https://ngdc.cncb.ac.cn/gsa/browse/CRA009110)^23^. The pipeline used for this analysis can download through Github platform (https://github.com/YongxinLiu/EasyAmplicon). Other data types, such as metadata, some taxonomic abundance datasets, and statistical results are accessible through Figshare platform. Visualizations of the analysis results have been provided as a part of this publication.

### Raw fastq data

The dataset are titled “16S rRNA gene sequence data from children with hand, foot and mouth disease and healthy individuals”. The dataset contains 1426 FASTQ file packages, with two files per sample (regarding forward and reverse reads). The purpose of sharing the FASTQ data is to allow other researchers to verify, reprocess and reanalyse the data according to their analytical needs and customized parameters.

### Sample metadata

Critical epidemiological information of the study population is included in the metadata to provide a reference for future retrospective analyses by other researchers. The metadata consists of demographic information (age, sex, group and address), type of enterovirus infection, hygienic habits, dietary habits (regularity of dietary habits and the frequency of probiotics, prebiotics and herbal tea intake) and information about the inserted primer sequences. Metadata for 713 participants and 458 participants were also provided as Figshare File 9^17^ and Figshare File 10^17^ to the article, respectively.

### Taxonomic abundance files and quality report

Statistical report of each sample sequence before merging were presented as File 1 in the Figshare. Taxonomy annotation tables for each ASV based on the RDP database can be accessed through File 2. And RDP classified taxonomic profile at five levels of taxonomic lineage is available in the files titled File 4 through 8.

## Technical Validation

The participant inclusion and exclusion criteria were strictly enforced, and the epidemiological information entry and stool collection processes for each subject were checked multiple times to ensure the authenticity and validity of the data.

The quality and concentration of extracted DNA were evaluated using a Thermo NanoDrop One instrument (Thermo Fisher Scientific, MA, USA). We verified the amplification length and concentration of the V4-1 region of the 16S rRNA gene by performing 1% agarose gel electrophoresis and visualizing the bands. Library quality was assessed on a Qubit Fluorometer (Thermo Fisher Scientific, MA, USA). Raw sequencing data quality was checked using FASTQC v0.11.9^24^ software after merging the forward and reverse reads, and the results showed that the quality score of each sample was above the Q20 reported accuracy, allowing further analyses. A total of 34,448,262 reads were identified among the 458 samples, with an average read length of 291.90 bases per sample. Quality control of merged sequences was performed using VSEARCH to control the expected allowed error in the amplicon sequence and obtain paired-end clean tags, resulting in 33,295,761 good quality reads. In addition, 565 ASVs were identified by USEARCH-based de novo classification, and 551 unique ASVs were screened by VSEARCH after removing unclassfied sequence.

**Fig. 2:**
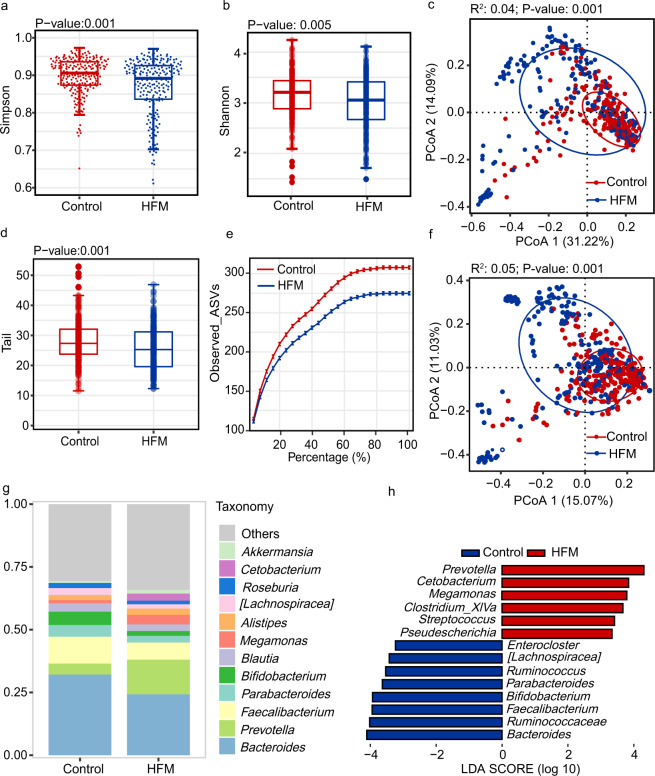
Characteristics of the gut microbiota profile in children with HFMD compared to that in healthy children. (**a**–**c**) Alpha diversity. Comparison the diversity of high-abundant bacteria (the Shannon and Simpson indice) and high-abundant bacteria (the Tail statistic) in the two groups (*P* < 0.05, Wilcoxon rank-sum tests). (**d**) Coverage of the detected faecal bacterial communities in the different groups. The red line represents the control group, and the blue line represents the HFMD group. Each vertical bar represents the standard error. (**e**,**f**) Principal coordinate analysis based on Bray‒Curtis distance and UniFrac distance showing patterns of separation in the bacterial communities between the two groups. PC1 and PC2 represent the top two principal coordinates, and the explanation of diversity is expressed as a percentage. Each point represents a single sample and is coloured based on the group. (**g**) Average relative abundance of the predominant bacterial taxa in each group at the genus level. (**h**) Differential bacterial taxonomy abundance between the groups was identified using linear discriminant analysis effect size (LEfSe) (LDA > 3). LDA, linear discriminant analysis.

Comparisons of microbiota composition and differences between the case and control groups was conducted based on the feature table. Differences in high-abundant bacteria between the HFMD group and the control group were observed in the alpha diversity analysis based on the Simpson and Shannon indices (Fig. 2a,b). Similarly, the Tail statistic analysis showed that the diversity of low-abundant bacteria was reduced in the case group compared to the control group (Fig. 2c). The rarefaction curve of the species richness indice at the ASV level reached an asymptote, which suggested that the sequencing depth captured most bacterial members in each group (Fig. 2d). Beta analysis based on the Bray‒Curtis distance and Unifrac distance with the Adonis test revealed that in the HFMD group, the bacterial microbiota profile was significantly dissimilar to that in the control group (Fig. 2e,f). To define the bacterial structures of the microbiomes in both groups, we created a bacterial stack plot at the genus level; the results indicated that *Bacteroides*, *Prevotella*, *Faecalibacterium*, *Parabacteroides*, and *Bifidobacterium* were the dominant genera (Fig. 2g). To further identify the bacteria that differed between the HFMD and control groups, differential abundance analysis using LEfSe (LDA score >4) confirmed that *Prevotella*, *Cetobacterium*, and *Megamonas* were enriched in the HFMD group, whereas HFMD-related genera, including *Bacteroides*, *Ruminococcus*, and *Faecalibacterium* were enriched in the control group (Fig. 2h).

## Supplementary information

Flow chart of subjects selection along with inclusion and exclusion criteria

The collection of Supplementary Figure 1 and Supplementary File 1

## Data Availability

Software versions used are all listed in the method descriptions. The data analysis pipeline for this study can available in GitHub (https://github.com/YongxinLiu/EasyAmplicon). And the custom code script of the Tail statistic analysis can be found in the Supplementary File 1.
