# Supplementary material for: A large scale 16S ribosomal RNA gene amplicon dataset of hand, foot and mouth patients and healthy individuals
Source: Sci Data. 2023 Jan 21;10:48. doi: 10.1038/s41597-023-01953-2 (PMC9867725; doi:10.1038/s41597-023-01953-2)
Supplement: Supplementary file 1 — Flow chart of subjects selection along with inclusion and exclusion criteria [file 41597_2023_1953_MOESM1_ESM.pdf]

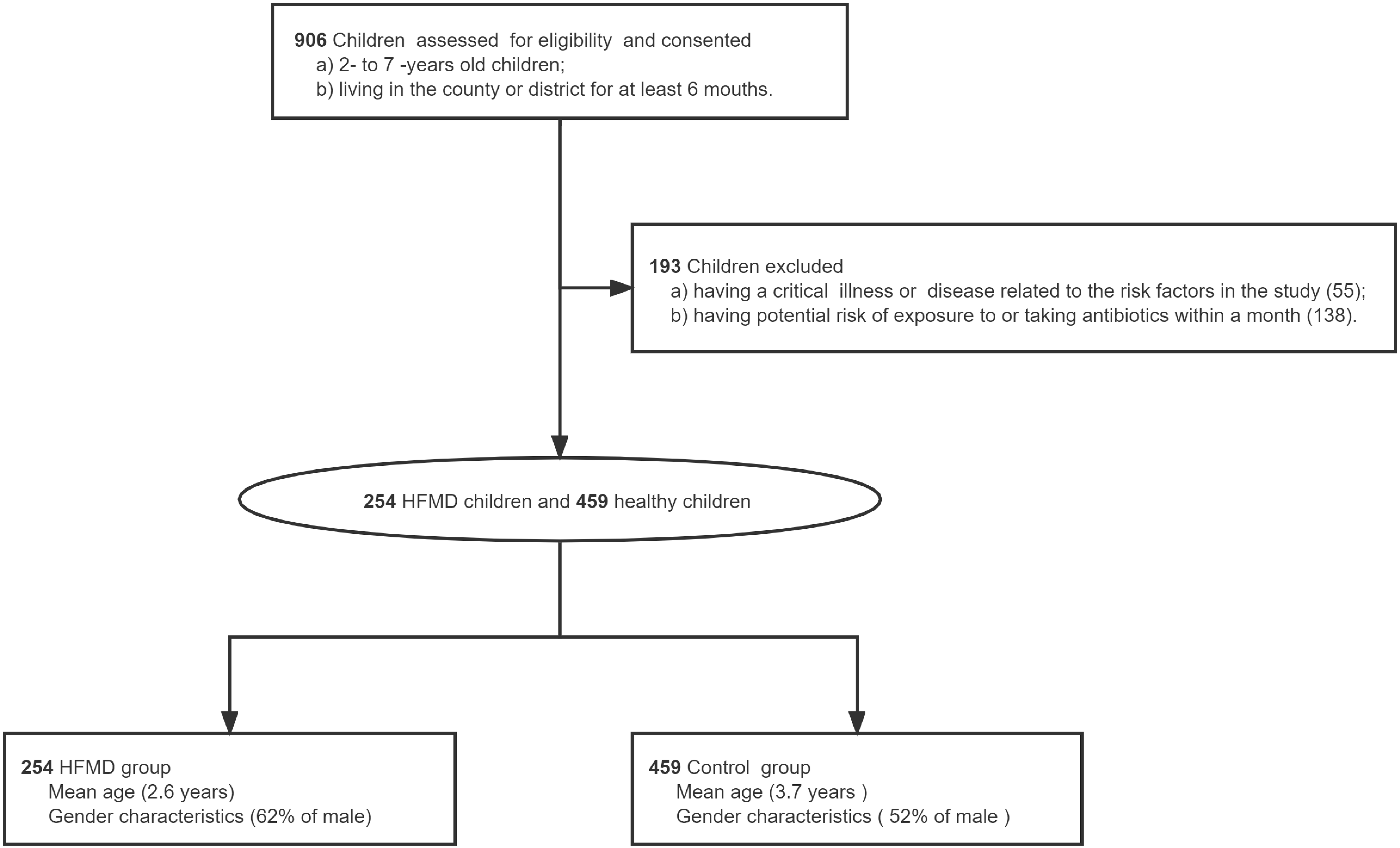
906 Children assessed for eligibility and consented
a) 2- to 7 -years old children;
b) living in the county or district for at least 6 mouths.
193 Children excluded
a) having a critical illness or disease related to the risk factors in the study (55);
b) having potential risk of exposure to or taking antibiotics within a month (138).
254 HFMD children and **459** healthy children
254 HFMD group
Mean age (2.6 years)
Gender characteristics (62% of male)
459 Control group
Mean age (3.7 years)
Gender characteristics (52% of male)
