# Supplementary material for: A large scale 16S ribosomal RNA gene amplicon dataset of hand, foot and mouth patients and healthy individuals
Source: Sci Data. 2023 Jan 21;10:48. doi: 10.1038/s41597-023-01953-2 (PMC9867725; doi:10.1038/s41597-023-01953-2)
Supplement: Supplementary file 2 — The collection of Supplementary Figure 1 and Supplementary File 1 [file 41597_2023_1953_MOESM2_ESM.pdf]

Contents

1. Supplementary Figures 2

1.1 Supplementary Figure 1 2

2. Supplementary File 3

2.1 Supplementary File 1 3

Supplementary Figures

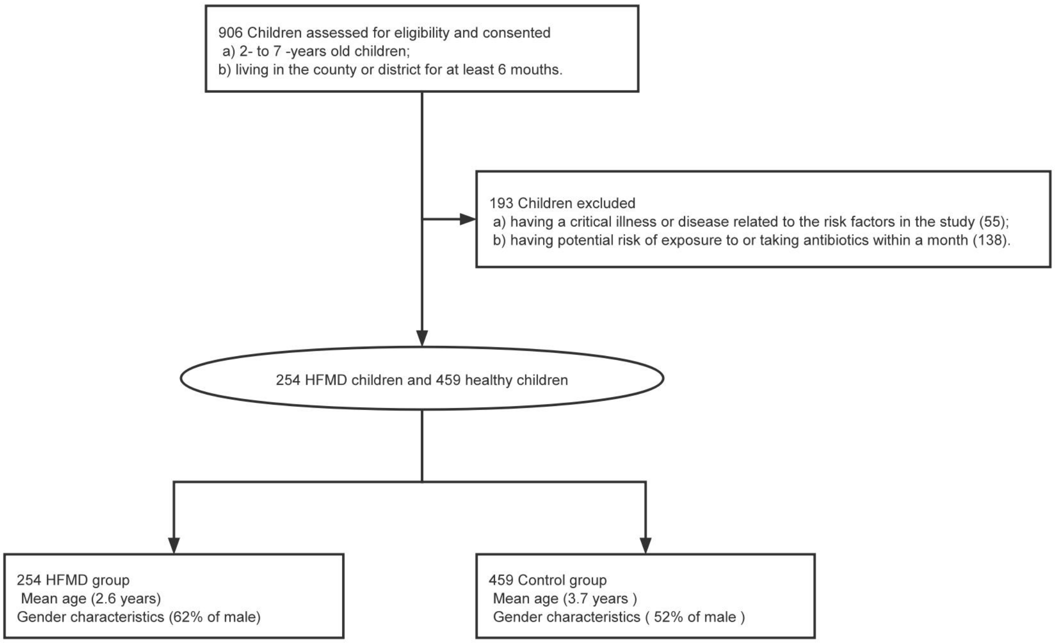

Supplementary Figure 1 Flow chart of subjects selection along with inclusion and exclusion criteria. 906 subjects who were assessed for eligibility and consented were included in the initial survey. After exclusion of health status and antibiotics usage history, 713 children were enrolled, including 254 hand-foot-mouth disease children (HFMD) and 459 healthy children. In the HFMD group, the average age of children was 2.6 years and males made up 62 percent of the group. In the control group, the average age of children was 3.7 years and 52 percent of them were male children.

Supplementary File

Supplementary File 1 The script of compute the Tail statistic index

```
#' tail_statistic
#' formula: sqrt(sigma(Pr[i]*(i-1)^2))
#' @param data otu/asv table
#' @return tail_statistic table
#' @export
#' @examples
#tail_statistic <- function(data) {

  stat <- lapply(data, function(x) {
    res <- sort(x[x>0], decreasing = T) / sum(x)
    index <- lapply(2:length(res), function(i) res[i]*(i-1)^2) %>%
      unlist() %>% sum() %>% sqrt()
  }) %>% as.data.frame %>% t %>% as.data.frame
  colnames(stat)[1] <- "Tail statistic"
  stat %>% tibble::rownames_to_column(var="sample.id")

}

#library(dplyr)
#data <- data.table::fread("otutab.txt", header=T) %>%
  tibble::column_to_rownames(var = "#OTUID")
#stat <- tail_statistic(data)
#openxlsx::write.xlsx(stat, "tail_statistic_result.xlsx", asTable = T)
```
